# *Acinetobacter baumannii* inhibits the PLK2/FoxO axis to induce oxidative stress and cell barrier dysfunction

**DOI:** 10.1128/spectrum.00694-26

**Published:** 2026-08-21

**Authors:** Jianxia Zhou, Lisha Huang, Fujian Zhou, Yajun Xiong, Xiaonan Zhang, Nannan Zhou, Congcong Shang, Yuying Feng, Xiaoya Niu, Yu Chen, Mengting Cai, Yingxin Chu, Jiayue Chen, Xiaojiang Zhang, Mingya Jiang, Yumiao Zhao, Aiguo Xu

**Affiliations:** 1 Department of Respiratory and Critical Care Medicine, The First Affiliated Hospital of Zhengzhou University 191599, Zhengzhou, China; 2 Tianjian Advanced Biomedical Laboratory, State Key Laboratory for Prevention and Treatment of Esophageal Cancer jointly established by the Ministry of Education, School of Medical Sciences, Zhengzhou University 12636, Zhengzhou, China; 3 Department of Cardiovascular Medicine, The Second Affiliated Hospital of Zhengzhou University 569063, Zhengzhou, China; Emory University School of Medicine, Atlanta, Georgia, USA

**Keywords:** *Acinetobacter baumannii*, FoxO pathway, PLK2, hypervirulent

## Abstract

*Acinetobacter baumannii* is a clinically prevalent opportunistic Gram-negative pathogen. Currently, hypervirulent *A. baumannii* strains have spread worldwide, while their molecular pathogenic mechanisms and effective therapeutic agents remain incompletely understood. This study aims to elucidate the core molecular mechanisms driving the pathogenesis of hypervirulent *A. baumannii*. Using transcriptomic profiling, we identified differentially expressed genes (DEGs) in human bronchial epithelial cells (BEAS-2B) infected with hypervirulent *A. baumannii*. In *in vitro* experiments, infection models were established using both BEAS-2B and HBE cell lines. The alterations in the PLK2 and FoxO signaling pathways were detected via western blot, qRT-PCR, and immunofluorescence assays. Intracellular oxidative stress levels were measured using dichlorodihydrofluorescein diacetate (DCFH-DA) probes. Transcriptomic analyses identified PLK2 as a key DEG, and the FoxO-mediated oxidative stress pathway was determined to be a key differentially regulated pathway after being infected by *A. baumannii*. Compared with the control group, the expression levels of PLK2, FoxO1, and FoxO3a were downregulated, intracellular ROS production was elevated, and the expression levels of barrier function-related proteins including ZO-1, Occludin, and E-cadherin were disrupted in the infection group. Inhibition or activation of PLK2 could, respectively, exacerbate and alleviate cell damage. In summary, this study reveals the effect of hypervirulent *A. baumannii* infection on PLK2 in host cells. It provides a new perspective for understanding the pathogenic mechanisms of hypervirulent *A. baumannii* and establishes an important theoretical basis for developing targeted therapeutic strategies in clinical practice. PLK2 may serve as a promising novel therapeutic target for hypervirulent *A. baumannii* infections.

**IMPORTANCE:**

This study provides crucial insights into the molecular pathogenesis of hypervirulent *A. baumannii* (HV *A. b*), a widespread and clinically challenging opportunistic pathogen. By establishing infection models in human bronchial epithelial cells (BEAS-2B and HBE) and performing transcriptomic analysis, the present study identified Polo-like kinase 2 (PLK2) as the most significantly dysregulated gene upon HV *A. b* infection. Furthermore, it revealed the critical involvement of the Forkhead box O (FoxO) signaling pathway and excessive intracellular oxidative stress in mediating bacterial pathogenesis. Key experimental findings demonstrate that HV *A. b* strains significantly suppress the protein levels of PLK2, FoxO1, and FoxO3a, thereby triggering intracellular ROS accumulation and impairing epithelial barrier function. The pivotal regulatory role of PLK2 uncovered in this work offers new perspective for understanding HV *A. b* virulence mechanisms. Importantly, this study identifies PLK2 as a highly promising novel therapeutic target for developing urgently needed targeted strategies against these difficult-to-treat infections.

## INTRODUCTION

*Acinetobacter baumannii* (*A. baumannii*) is a Gram-negative pathogen capable of causing severe hospital-acquired infections and is widely recognized for its extensive drug resistance. This pathogen can not only colonize and persist in hospital environments for prolonged periods, but it is also resistant to various adverse conditions, including antibiotic pressure, nutrient deprivation, and environmental stressors (1). The genome of this bacterium exhibits high plasticity, which drives the emergence of increasingly threatening drug-resistant strains. Consequently, owing to its significant public health implications, multidrug-resistant *A. baumannii* has been classified as a critical global health threat by major international health authorities, such as the European Centre for Disease Prevention and Control (ECDC), the Infectious Diseases Society of America (IDSA), and the World Health Organization (WHO) (2).

Hypervirulent *A. baumannii* (HV *A. b*) strains possess strong environmental adaptability, robust biofilm formation ability, and the capacity to cause invasive infections in immunocompetent hosts, posing a clinical challenge that needs to be addressed (3). Nevertheless, accumulating research on the pathogenicity of *A. baumannii* and the development of non-antibiotic therapeutic strategies indicates that virulence represents a crucial pathogenic trait that cannot be neglected. The development of novel non-antibiotic interventions targeting virulence mechanisms has emerged as a core research direction for mitigating the pathogenicity of this pathogen (4–6). Therefore, systematic investigation of the molecular pathogenic mechanisms and identification of upstream regulatory targets for intervention are of important theoretical and clinical significance.

Reports suggest that most bacteria, including *A. baumannii*, ensure long-term survival via the rapid acquisition of novel phenotypes—such as altered gene expression or activation of specific signaling pathways—in response to external stress (7). For example, under prolonged antibiotic exposure, *A. baumannii* can acquire drug resistance to escape the bacteriostatic or bactericidal activities of antibiotics (8). However, while the majority of these studies focus on phenotypic and genotypic changes within the bacteria themselves, few investigate host adaptation mechanisms through the lens of host-pathogen interactions.

The Polo-like kinases (PLKs) constitute a serine/threonine kinase family comprising five mammalian members: PLK1, PLK2/SNK, PLK3/FNK, PLK4/SAK, and PLK5 (9). Among these, the C-terminal Polo-box domain serves as the core structural module mediating protein-protein interactions (10). Accumulating evidence indicates that PLK2 plays a prominent role in antioxidant defense. In particular, it decreases reactive oxygen species (ROS) levels by enhancing NRF2 activity, thereby alleviating oxidative stress-induced damage and cell death (11). Beyond its antioxidant function, PLK2 has been implicated in regulating the release of *Avian metapneumovirus Type C* (*aMPV/C*) from Vero cells (12). Furthermore, by targeting viral surface proteins, PLK2 modulates early-stage viral replication within host cells (13), providing new insights for mitigating host cell responses after infection and developing antiviral therapies. To date, research on PLK2 in infectious disease contexts remains limited. While current studies largely focus on its role in regulating viral infections, its regulatory function in host responses to bacterial infections, particularly those caused by *A. baumannii*, remains unclear.

The FoxO transcription factor family constitutes a critical component of the mammalian antioxidant defense system. It comprises four members, namely, FoxO1, FoxO3, FoxO4, and FoxO6. FoxO1, 3, and 4 exhibit extensive and overlapping expression in both developing and mature tissues, whereas FoxO6 is predominantly expressed in specific brain regions (14). As key regulators of cellular stress responses, FoxOs exert antioxidant effects by regulating antioxidant enzymes (e.g., MnSOD and catalase) and reducing intracellular ROS accumulation (15). The activity of FoxOs is strictly regulated via post-translational modifications, including acetylation, ubiquitination, and phosphorylation. Such modifications reshape cellular transcriptional processes and thereby modulate downstream mechanisms (16). Studies have shown that pathogenic infection suppresses the activity of serine/threonine kinase AKT and drives nuclear translocation of FoxOs. In addition, FoxOs preserve intestinal microbiota homeostasis by stimulating the secretion of antimicrobial peptides (17). Our previous work also demonstrated that SIRT1 upregulates FoxO1 expression, which alleviates *A. baumannii*-induced cell damage by enhancing antioxidant enzyme activity and reducing ROS release. However, the relationship between PLK2 and the FoxO signaling axis, as well as its regulatory role in the pathogenicity of HV *A. b*, remains unclear.

In this study, we identified a regulatory relationship between PLK2 and the FoxO pathway. BEAS-2B and HBE cell models infected by HV *A. b* were established. Our results reveal that hypervirulent strains significantly downregulate the expression of PLK2 and FoxO, while activating oxidative stress and inducing barrier dysfunction. Inhibition of PLK2 aggravates the damaging effect of HV *A. b*. In summary, our findings demonstrate that PLK2 modulates oxidative stress and barrier damage by regulating the FoxO antioxidant pathway. This indicates that PLK2 may serve as a novel target for suppressing the pathogenicity of HV *A. b*.

## MATERIALS AND METHODS

### Cultivation of BEAS-2B cells, HBE cells, and *A. baumannii* strains

BEAS-2B and HBE cells were purchased from Servicebio Biotechnology Co., Ltd. (Zishan biotech, China). The *A. baumannii* strains (AB5075 and ATCC 19606) were gifted to us by the respiratory lab of our hospital. BEAS-2B and HBE cells were passaged every 3 days and cultivated in RPMI 1640 medium (Wuhan Procell Biotechnology Co., Ltd.) with 10% Fetal Bovine Serum (FBS, Keygen BioTECH, KGL3006) and streptomycin (huayunbio, B3034EA) at 37°C and 5% CO_2_. The type strain AB5075 serves as a model strain characterized by stable virulence expression and excellent genetic operability. It is frequently utilized as a model strain for exploring the pathogenic mechanism of *A. baumannii*. The type strain ATCC 19606 was employed as a control for low virulence *A. baumannii* (LV *A. b*) in the present study. Strains stored in an ultra-low temperature freezer at −80°C were thawed on ice under sterile conditions. A volume of 50 μL bacterial suspension was transferred to Luria-Bertani (LB, HKM) medium and spread evenly. The plates were incubated at 37°C for 12 h. Single colonies were picked from the plate and inoculated into liquid culture medium, which was then shaken at 37°C at 200 rpm. Bacteria in the logarithmic growth phase were utilized for infection.

### Establishment of cell infection models

To establish infection models, *A. baumannii* in the logarithmic growth phase was washed twice with sterile PBS and resuspended in antibiotic-free RPMI 1640 complete medium. Bacterial concentration was adjusted to a multiplicity of infection (MOI) of 10. BEAS-2B or HBE cells in the logarithmic growth phase were inoculated into 6-well plates. When the cell confluence reached approximately 80%–90%, the original medium was removed, and fresh medium containing the *A. baumannii* suspension at an MOI of 10 was added to facilitate the co-culture of bacteria and host cells. Infections were performed in a 37°C with 5% CO₂ incubator for a total period of 6 h. No washing or antibiotic treatment was applied to eliminate uninternalized extracellular bacteria after infection, which aimed to simulate and observe the comprehensive host responses triggered by bacterial exposure.

### Transcriptional and bioinformatic analysis

The RNA-seq data used in this study were generated in our previous study (18). In brief, BEAS-2B cells were infected with hypervirulent *A. baumannii* for 6 h and subjected to transcriptomic analysis. Four biological replicates were set up for each group. Total RNA from BEAS-2B cells was extracted using the SteadyPure Universal RNA Extraction Kit II (Accurate Biotechnology [Hunan] Co., Ltd., Changsha, China, AG21022). Library preparation and sequencing were performed by Guangzhou Aozhi Biotechnology Co., Ltd. Genes with a log₂ |Fold Change| > 1 and an adjusted *P-*value (FDR) < 0.05 were regarded as differentially expressed genes (DEGs). Volcano plots and heatmaps were generated in R using the ggplot2 and pheatmap packages, respectively. Functional enrichment analyses, GO enrichment, KEGG pathway analysis, and GSEA were also performed using the R packages.

### Western blot

Cells were lysed in Radio Immunoprecipitation Assay buffer (RIPA, Proteintech), supplemented with phenylmethylsulfonyl fluoride (PMSF, Solarbio), and protease inhibitor (MCE, HY-K0010) for 30 min, followed by centrifugation at 12,000 *g* for 15 min. Proteins were separated by SDS-PAGE (Vazyme) and transferred onto a PVDF membrane (Millipore). After blocking with 5% BSA, the membrane was incubated with primary antibodies (GAPDH, 1:5,000, zen-bioscience; FoxO1, 1:1,000, Affinity biosciences; FoxO3a, 1:1,500, zen-bioscience, R381451; PLK2, 1:500, OriGene, TA380043S; E-cadherin,1:1,000, HUABIO, ET1607-75; ZO-1, 1:1,000, zen-bioscience, 511417; Occludin, 1:1,000, MCE, HY-P81155) and a secondary antibody (rabbit, 1:5,000, Abmart). Signals were detected with a chemiluminescence imaging system and quantified using ImageJ.

### Immunofluorescence

BEAS-2B cells were fixed using 4% paraformaldehyde (Biosharp) for 20 min and washed with PBS three times. Cells were then permeabilized with 0.1% Triton X-100 (Solarbio, T8200) for 10 min and blocked with 5% BSA (Biofroxx, 4240GR100) for 1 h. The primary antibodies were added, and the cells were incubated overnight on a shaker at 4°C. The following day, the cells were incubated with fluorescent secondary antibodies, followed by staining with DAPI (Beyotime). Signals were visualized under an inverted fluorescence microscope and quantified using ImageJ.

### Cytotoxicity and MOI testing

Cytotoxicity was determined using the CCK-8 assay kit (Beyotime). BEAS-2B cells at a density of 5 × 10^3^ per well were seeded into 96-well plates (NEST Biotechnology Co., Ltd. [Wuxi, China]) and cultured in 100 μL RPMI medium. After treatment, 10 μL CCK-8 working solution was added to each well, followed by incubation at 37°C for 2 h in the dark. Absorbance at 450 nm was measured using a microplate reader (Bio-Rad). The MOI is defined as the ratio of bacterial colony-forming units (CFU) to the number of host cells. An MOI of 10 was adopted in this study, as preliminary experiments confirmed that this ratio could achieve a high cell infection rate while avoiding excessive toxicity.

### Measurement of reactive oxygen species

ROS levels were measured with dichlorodihydrofluorescein diacetate probes (DCFH-DA, Solarbio). Cells were incubated with 10 μM dissolved in DMSO (Solarbio) at 37℃ for 30 min under dark conditions. After washing three times with PBS, fluorescence signals were captured via fluorescence microscopy, and the signal intensity was subsequently quantified with ImageJ software.

### Test for functional permeability (FITC-dextran)

A total of 3 × 10^4^ BEAS-2B cells were seeded in 600 μL complete medium in the upper compartment of Transwell inserts placed in 24-well plates, with 1 mL medium added to each lower compartment. After grouping and treatment, medium in the upper compartment was removed, and inserts were washed with PBS. A total volume of 600 μL of FITC-dextran working solution (0.5 mg/mL in PBS, Beyotime) was added to the upper compartment, and plates were incubated in the dark for 30 min. Finally, 100 μL aliquots was collected and analyzed using a microplate reader (excitation = 492 nm, emission = 520 nm) to calculate the permeability index (Pa/Pc%).

### Quantitative real-time PCR

RNA was extracted with the *SteadyPure* Universal RNA Extraction Kit II in strict accordance with the provided instructions. The Prime Script RT reagent kit (GOONIE) was used for complementary DNA (cDNA) synthesis. A 20 μL reaction system was prepared in PCR tubes (Bioland, China), consisting of 1 μL of cDNA, 1 μL of forward primers, 1 μL of reverse primers, 7 μL of DEPC water (TargetMol), and 10 μL of SYBR qPCR Master Mix (Vazyme). GAPDH was used as an internal control, and the primer sequences are presented in Table S1.

### Statistical analysis

Data are presented as the mean ± standard error of the mean (SEM). Statistical significance was determined by Student’s *t*-test (for comparisons between two groups) and ANOVA (for comparisons among more than two groups). All experiments were performed at least three independent times, and the results were analyzed and plotted using GraphPad Prism 9. Statistical significance was set at *P* < 0.05.

## RESULTS

### Transcriptional alterations in BEAS-2B cells infected by HV *A. b*

**Fig 1:**
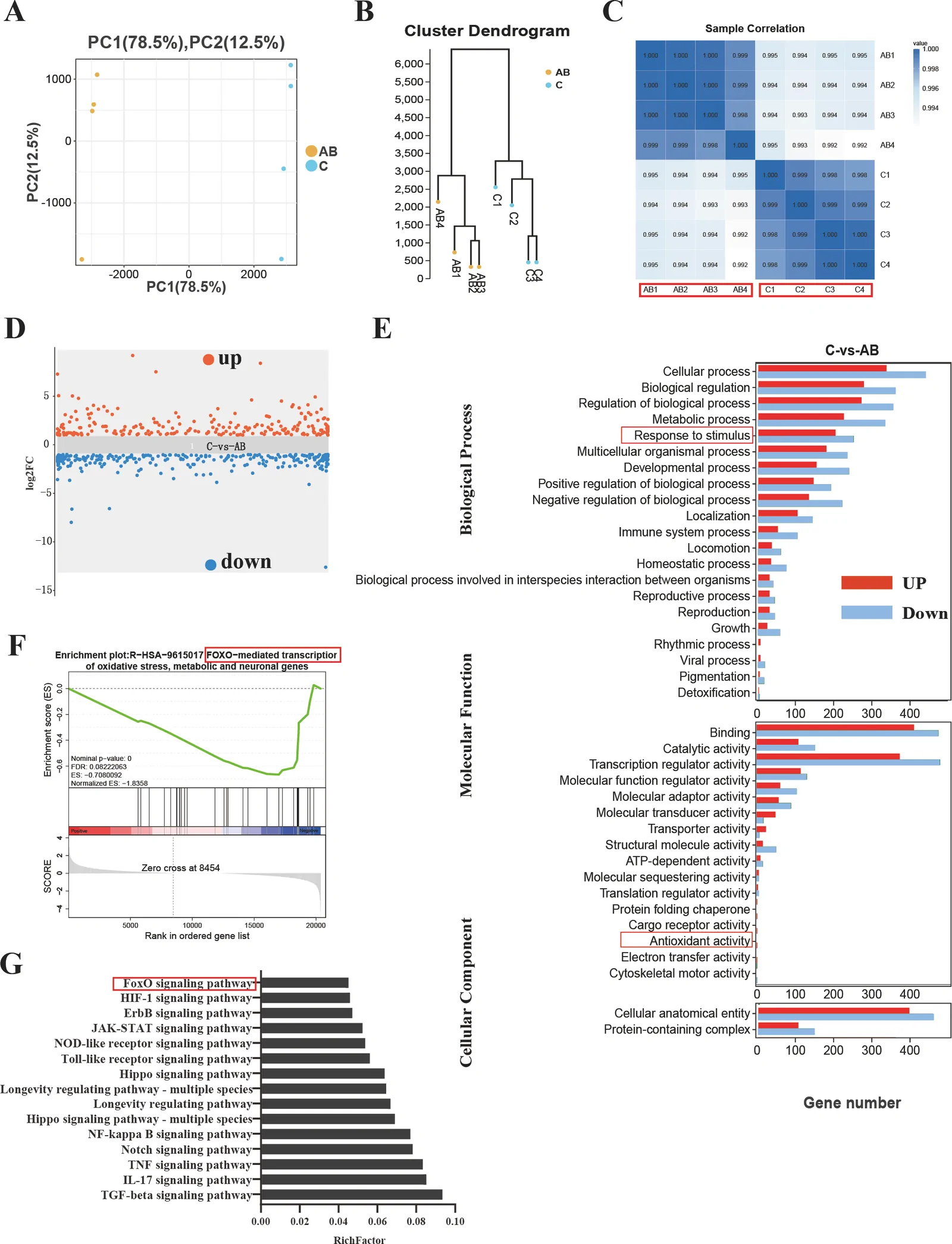
Transcriptional profiling of BEAS-2B cells infected with HV *A. b* strains. (**A**) PCA of globally normalized gene expression profiles. Each dot represents a biological replicate, colored by group (C, control; AB, HV *A. b* infection). (**B**) Hierarchical clustering dendrogram based on Euclidean distance and complete linkage. Branch arrangement reflects transcriptional similarity among samples. (**C**) Pairwise Pearson correlation matrix of normalized expression values. Color intensity corresponds to correlation coefficient (range, 0–1; AB1–AB4, HV *A. b*-infected biological replicates; C1–C4, uninfected controls; *n* = 4). (**D**) Volcano plot DEGs. Red and blue dots denote upregulated and downregulated genes. (**E**) GO enrichment analysis of DEGs, including BP, CC, and MF. Bar length is proportional to gene count. (**F**) GSEA confirming the perturbation of the FoxO-mediated oxidative stress response pathway. (**G**) KEGG pathway enrichment analysis highlighting key dysregulated signaling cascades.

Following a comprehensive assessment of bacterial infection efficiency and post-infection cell viability, BEAS-2B cells were infected with a log-phase bacterial suspension at an MOI of 10. Samples were collected for analysis 6 h post-infection (Fig. S1A and B). Three complementary analytical approaches were applied to assess transcriptome repeatability and transcriptional divergence between groups. Principal component analysis (PCA; Fig. 1A) and a hierarchical clustering dendrogram (Fig. 1B) reflected global transcriptional differences among all samples. Based on infection status, the control group (C1, C2, C3, C4) and the infected group (four replicates infected by AB5075 strains) were clearly clustered into two distinct clusters, with consistent clustering results between the two analyses. The sample correlation checkerboard heatmap (Fig. 1C) further quantified pairwise similarity via Pearson correlation coefficients. Both groups exhibited strong inter-group correlation values (*r* = 0.99–1), verifying excellent consistency within biological replicates. Collectively, these multidimensional analyses indicate that AB5075 infection induced distinct transcriptional phenotypes in host cells. DEGs were screened according to strict thresholds: FDR < 0.05 and *P* < 0.05. A total of 483 significant DEGs were identified, including 222 upregulated and 261 downregulated genes in infected cells compared with the control group (Fig. 1D).

Furthermore, Gene Ontology (GO) and Kyoto Encyclopedia of Genes and Genomes (KEGG) enrichment analyses were performed. Although the analyses identified a wide range of significantly enriched biological processes, the discussion focused on the categories most directly relevant to oxidative stress, FoxO signaling, and epithelial barrier dysfunction, which were the central themes of the experimental investigations and validations. GO enrichment, which was classified into biological process (BP), molecular function (MF), and cellular component (CC), indicated that DEGs were highly enriched in BP terms governing biological processes regulation, metabolic reactions, and cellular responses to external stimuli. Meanwhile, MF terms included antioxidant stress responses and ATP-dependent activities. This finding led us to conclude that *A. baumannii* infection impairs cellular compensatory responses to external stimuli (Fig. 1E). We next performed KEGG pathway enrichment analysis and filtered pathways with a Rich Factor > 0.05, yielding 15 classical signaling pathways, among which the FoxO signaling pathway (regulating cell metabolism, proliferation, and oxidative stress) was prominently enriched (Fig. 1G). To evaluate global biological changes independent of differential gene selection, Gene Set Enrichment Analysis (GSEA) was conducted on the entire gene set. Pathways were defined as significantly enriched when satisfying following thresholds: absolute Normalized Enrichment Score (NES) > 1, FDR < 0.25, and *P* < 0.05. GSEA confirmed significant suppression of the FoxO-mediated pathway in infected cells relative to controls (Fig. 1F).

Therefore, genes involved in FoxO-dependent antioxidant stress and metabolic homeostasis were downregulated in HV *A. b-*infected cells compared with controls, implying that HV *A. b* impairs cellular antioxidant function. These findings suggest that the inactivation of the FoxO-mediated antioxidant signaling pathway plays a crucial role in promoting HV *A. b*-induced cell damage.

### HV *A. b* inhibits PLK2 and FoxO-mediated antioxidant stress pathway

**Fig 2:**
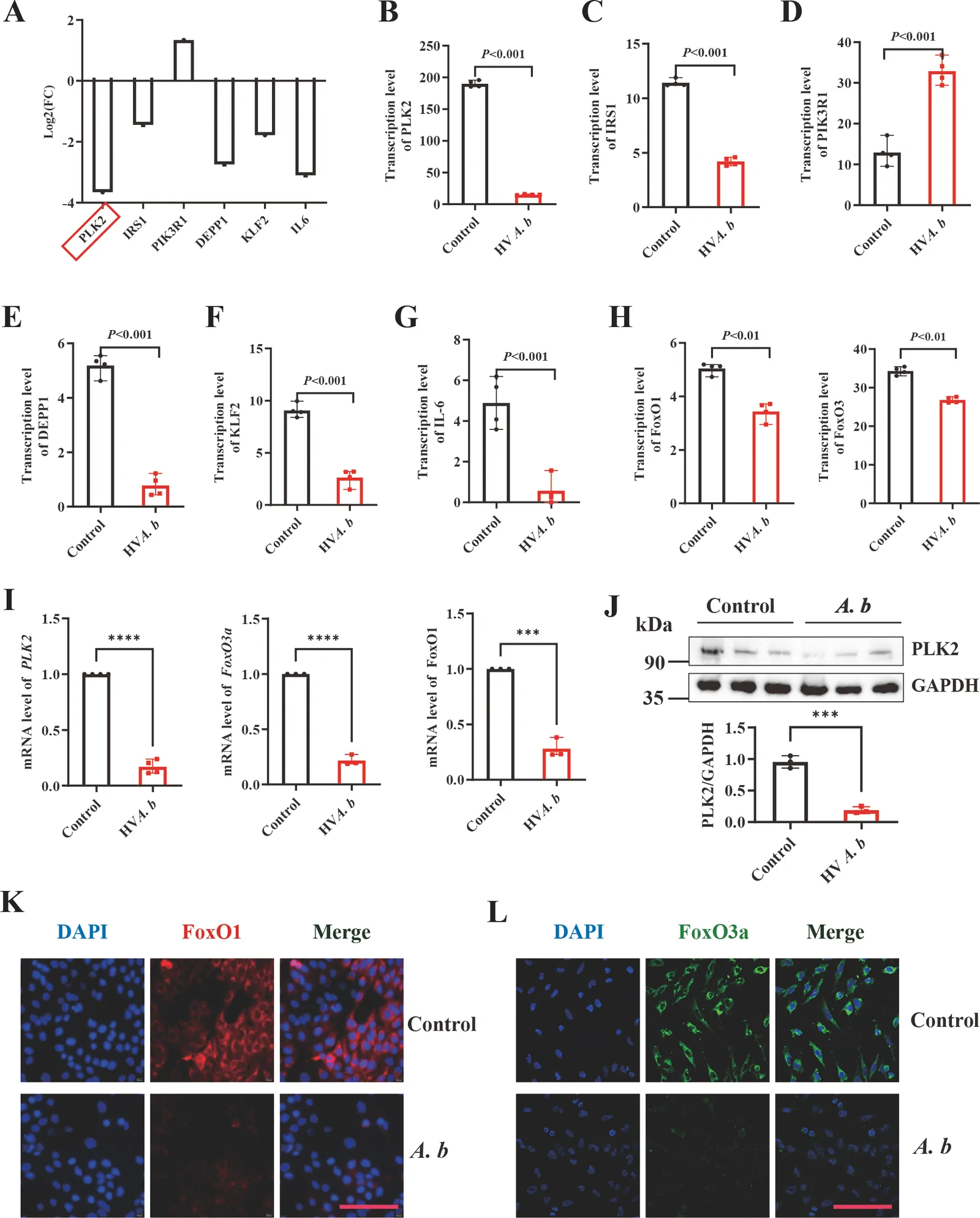
HV *A. b* downregulated the expression levels of PLK2, FoxO3a, and FoxO1. (**A**) Key genes involved in FoxO pathway identified from transcription results. (**B through G**) Transcriptional abundance of *PLK2, IRS1, PIK3R1, DEPP1, KLF2,* and *IL6*. (**H**) Transcript levels of FoxO1 and FoxO3a. (**I**) The mRNA levels of *PLK2, FoxO3a*, and *FoxO1* quantified via qRT-PCR (*n* = 3). (**J**) The protein levels and quantitative results of PLK2 (*n* = 3). (**K and L**) Representative immunofluorescence images of FoxO1 and FoxO3a. Images of DAPI (blue), FoxO1 (red), and FoxO3a (green) were acquired under an inverted fluorescence microscope (Scale bar: 80 μm). ***P < 0.001, ****P < 0.0001.

To expand on our prior findings, we conducted a more comprehensive and in-depth exploration of the FoxO pathway and its upstream regulatory targets. Transcriptomic analysis identified PLK2, IRS1, PIK3R1, DEPP1, KLF2, and IL-6 as the top DEGs linked to the FoxO pathway (*P* < 0.01; Fig. 2A). Notably, PLK2 showed the most striking difference, with its transcript level decreasing by approximately 10-fold upon infection (Fig. 2B), while other FoxO pathway genes showed relatively moderate changes (two- to fivefold upregulated or downregulated; Fig. 2C through G). Transcriptome analysis also revealed reduced expression of *FoxO1* and *FoxO3a* in infected cells (Fig. 2H). We further verified the expression levels of *PLK*2, *FoxO*1, and *FoxO*3*a* via qRT-PCR. The results indicate that HV *A. b* reduced their intracellular mRNA levels by approximately 5- to 10-fold, consistent with the RNA-seq data (Fig. 2I). PLK2 protein level was further identified by western blot (Fig. 2J). IF staining was performed to verify the nuclear localization and fluorescence intensity of FoxO1 and FoxO3a (Fig. 2K and L). We observed weaker fluorescent signals of FoxO1 and FoxO3a in infected cells. Collectively, these data suggest that both *PLK2* and FoxO-mediated antioxidant stress pathways are inhibited upon HV *A. b* infection. It is hypothesized that PLK2 mediates HV *A. b*-induced intracellular oxidative stress by regulating the FoxO pathway.

### PLK2 regulates intracellular antioxidant capacity mediated by FoxO1 and FoxO3a

**Fig 3:**
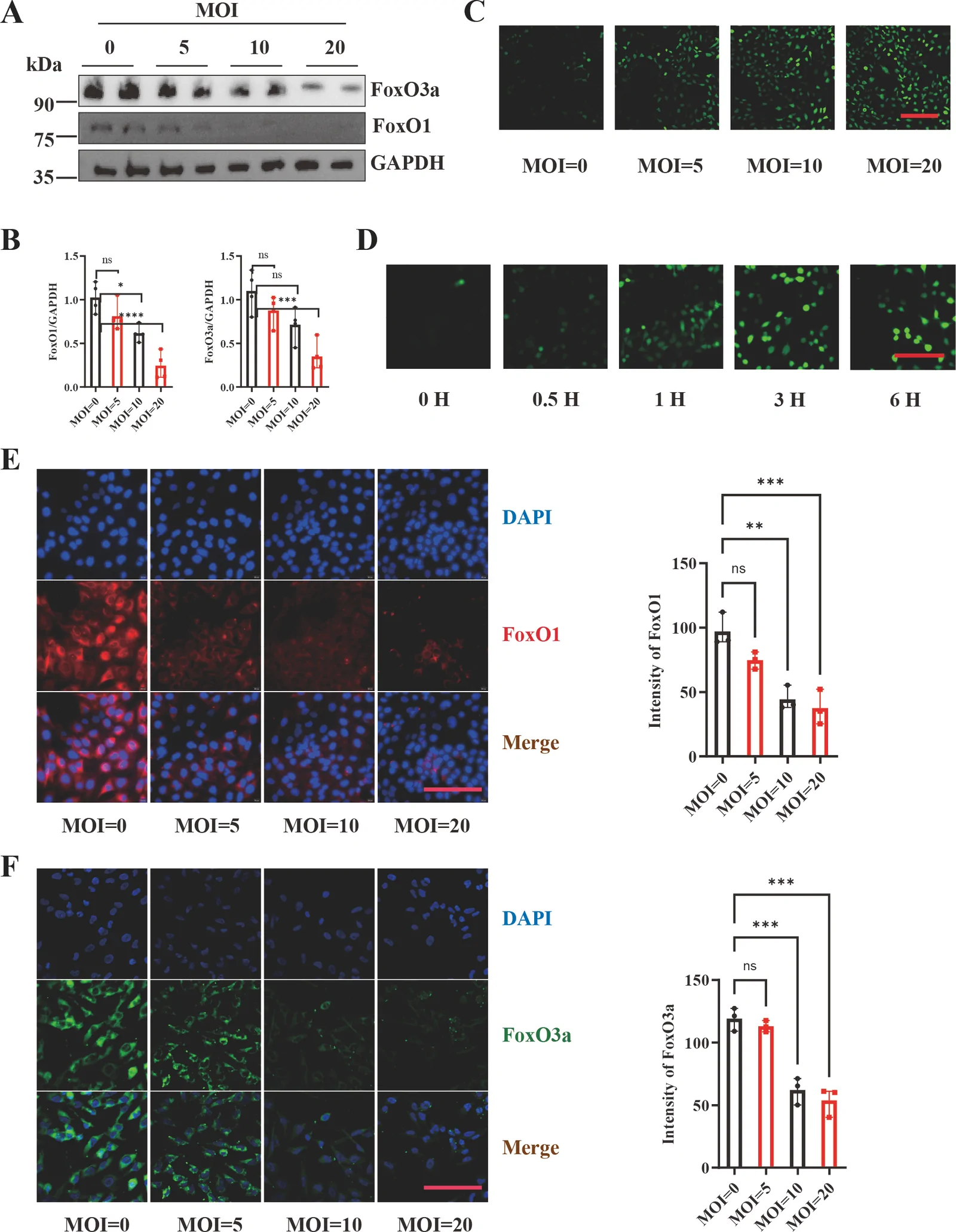
HV *A. b* downregulates PLK2, FoxO3a, and FoxO1 expression in a dose-dependent manner. (**A and B**) Protein levels and quantitative analysis results of FoxO1 and FoxO3a at MOIs of 0, 5, 10, and 20. (**C**) Intracellular ROS accumulation at different MOIs measured with DCFH-DA probes (Scale bar: 100 μm). (**D**) Time-dependent ROS accumulation detected via DCFH-DA probes (Scale bar: 50 μm). (**E and F**) Representative immunofluorescence images and quantitative results of FoxO1 and FoxO3a. DAPI (blue) and FoxO1 (red)/FoxO3a (green) were detected by inverted fluorescence microscope (Scale bar: 80 μm). ns: no significance. *P < 0.05, **P < 0.01, ***P < 0.001, ****P < 0.0001.

BEAS-2B cells were incubated with increasing concentrations of HV *A. b* suspensions, and the intracellular levels of antioxidant transcription factors FoxO1 and FoxO3a were quantified. Western blot assays revealed that HV *A. b* suppressed FoxO1 and FoxO3a expression in a dose-dependent manner. At an MOI of 10 or 20, FoxO1 and FoxO3a protein levels decreased significantly (*P* < 0.05; Fig. 3A and B). Simultaneously, as bacterial dosage and infection duration increased, ROS accumulation rose accordingly. ROS levels markedly elevated when the MOI exceeded 10 (Fig. 3C). Intracellular ROS accumulated rapidly at 1 h post-infection, peaked at 3 h, and remained a high level at 6 h (Fig. 3D). In addition, immunofluorescence staining was performed to detect the expression and subcellular localization of FoxO1 and FoxO3a. Higher infection MOIs led to a prominent reduction in FoxO1 and FoxO3a fluorescent signals (Fig. 3E and F). These data demonstrate that HV *A. b* impairs the antioxidant capacity of BEAS-2B cells.

**Fig 4:**
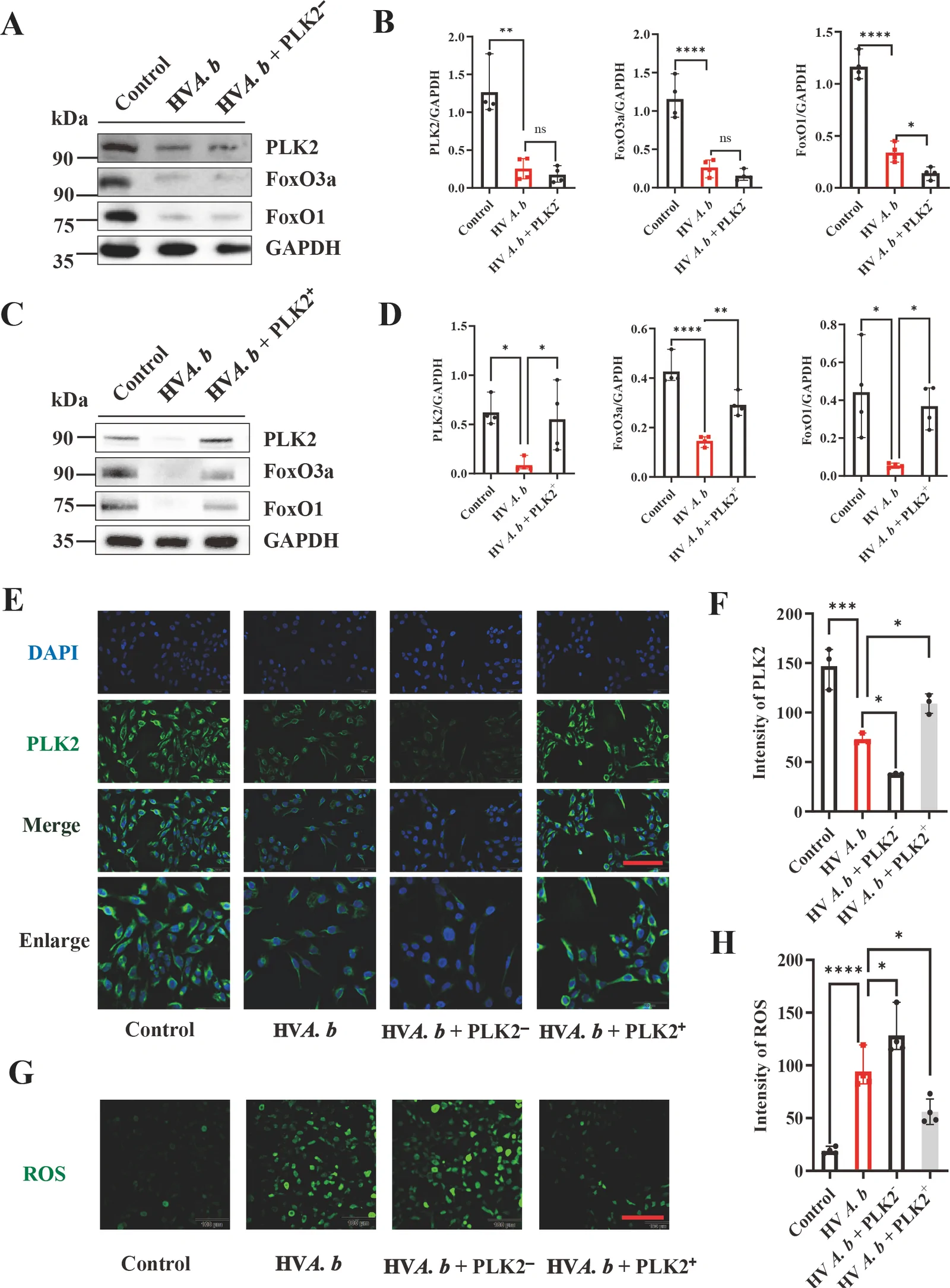
PLK2 modulates FoxO-dependent antioxidant stress responses. (**A through D**) Protein levels and quantitative results of PLK2, FoxO1, and FoxO3a (*n* =4). (**E and F**) Representative fluorescence images of PLK2 and corresponding quantitative analysis (*n* = 3, Scale bar: 100 μm). (**G and H**) Intracellular ROS accumulation tested by DCFH-DA probes and quantitative fluorescence analysis (Scale bar: 100 μm). ns: no significance. *P < 0.05, **P < 0.01, ***P < 0.001, ****P < 0.0001.

PLK2 was inhibited or activated to dissect its role in the regulation of antioxidant capacity. Compared with the infection group, PLK2 suppression further downregulated FoxO1 and FoxO3a protein expression (Fig. 4A and B). In contrast, PLK2 activation restored FoxO1 and FoxO3a abundance (Fig. 4C and D), confirming a functional link between PLK2 and FoxO-mediated antioxidant defense. Immunofluorescence further demonstrated that PLK2 inhibitor and agonist markedly altered the fluorescence intensity of PLK2 (Fig. 4E and F). We also measured ROS levels and found they correlated closely with PLK2 activity (Fig. 4G and H). PLK2 activation effectively alleviated excessive ROS accumulation, establishing a reciprocal relationship between PLK2 activity, FoxO function, and oxidative stress.

### PLK2 repairs the cell barrier function damaged by HV *A. b*

**Fig 5:**
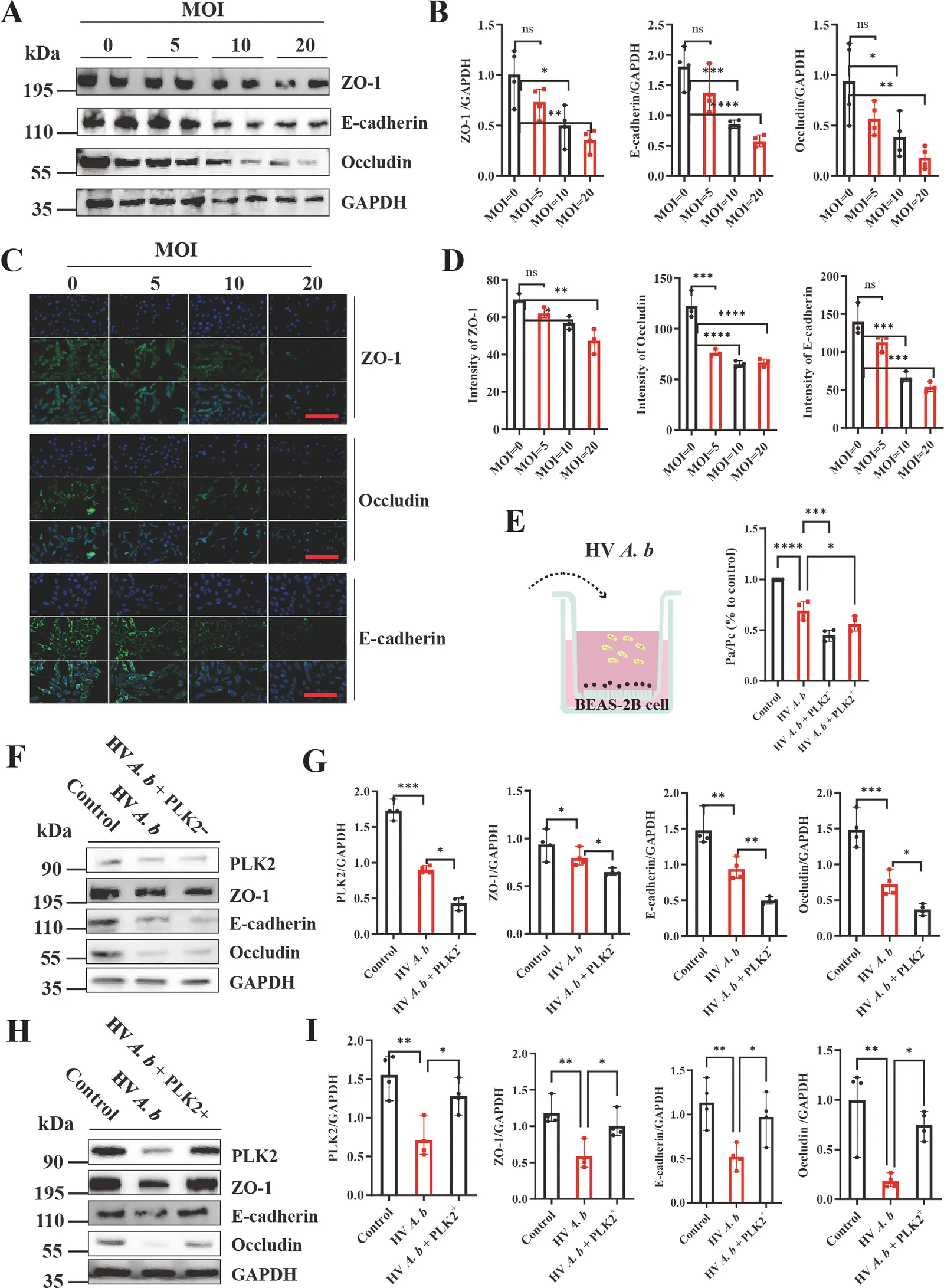
PLK2 regulates epithelial barrier function to mitigate HV *A. b*-induced cell damage. (**A and B**) Protein levels and quantitative results of ZO-1, E-cadherin, and Occludin (*n* = 4). (**C**) Representative fluorescence images of ZO-1, E-cadherin, and Occludin at different MOIs (0, 5, 10, 20) (Scale bar: 50 μm). (**D**) Quantitative assessment of the fluorescence intensity of ZO-1, E-cadherin, and Occludin (*n* = 3). (**E**) FITC-dextran permeability assay for evaluating epithelia barrier damage (*n* = 4). (**F through I**) Protein levels and quantitative results of PLK2, ZO-1, E-cadherin, and Occludin (*n* = 4). ns: no significance. *P < 0.05, **P < 0.01, ***P < 0.001, ****P < 0.0001.

Epithelial barrier integrity was assessed by quantifying the expression of tight junction proteins, including ZO-1, Occludin, and E-cadherin. Western blot analysis revealed that HV *A. b* infection dose-dependently downregulated the expression of these proteins (Fig. 5A and B). Consistent with this, immunofluorescence staining demonstrated that increasing the MOI led to pronounced intercellular gaps and diminished fluorescence intensity of ZO-1, Occludin, and E-cadherin (Fig. 5C and D). Functional permeability assays further revealed that PLK2 inhibition exacerbated HV *A. b-*induced barrier impairment, whereas PLK2 activation markedly alleviated barrier leakage (Fig. 5E). At the molecular level, PLK2 suppression further diminished the protein levels of ZO-1, Occludin, and E-cadherin compared to the infection group (Fig. 5F and G), while the activation of PLK2 partially restored their expression (Fig. 5H and I). Collectively, given the reduced PLK2 fluorescence and increased ROS accumulation upon PLK2 inhibition, these observations indicate that PLK2 exerts a protective effect against epithelial barrier dysfunction induced by HV *A. b*.

### PLK2 mitigates oxidative stress and epithelial barrier dysfunction in HBE cells

**Fig 6:**
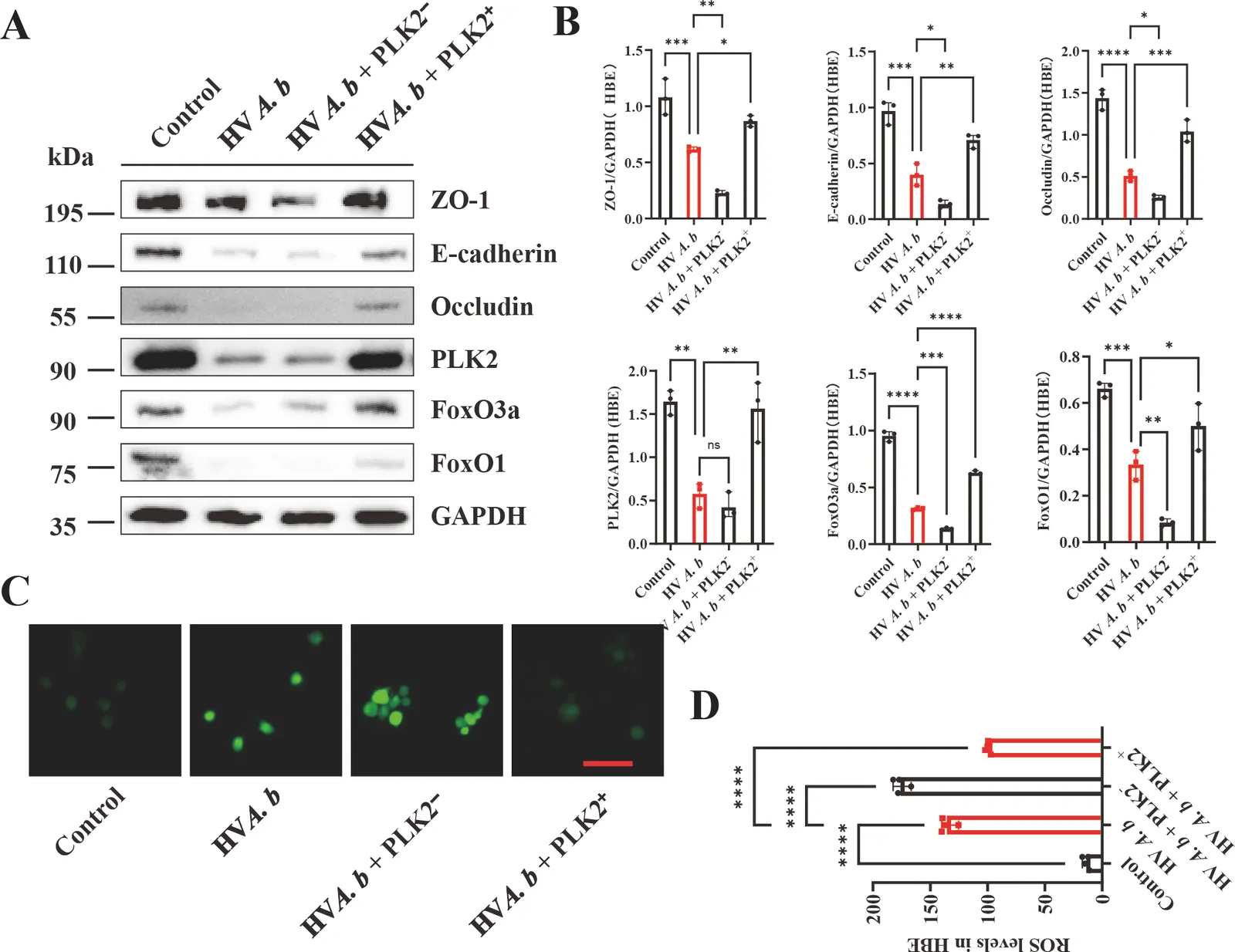
HV *A. b* induces damage to HBE cells and disrupts barrier function by inhibiting PLK2. (**A**) Protein levels of ZO-1, E-cadherin, Occludin, PLK2, FoxO1, and FoxO3a. (**B**) Quantitative protein results of ZO-1, E-cadherin, occludin, PLK2, FoxO1, and FoxO3a (*n* = 3). (**C**) Intracellular ROS levels in HBE cells tested by DCFH-DA probes. (**D**) Quantitative results of ROS (*n* = 4). ns: no significance. *P < 0.05, **P < 0.01, ***P < 0.001, ****P < 0.0001.

To confirm whether PLK2-mediated cytoprotection represents a distinct defensive mechanism against hypervirulent strains, we utilized an HBE cell infection model as described previously. Four experimental groups were set up as before. Comparative analysis showed that HV *A. b* infection significantly suppressed the expression of tight junction proteins (ZO-1, E-cadherin, and Occludin) and key antioxidant regulators (FoxO1 and FoxO3a) (Fig. 6A and B). Conversely, PLK2 activation effectively decreased these detrimental effects and reduced HV *A. b-*induced ROS accumulation (Fig. 6C and D). Additionally, while low virulence strains triggered a comparable increase in ROS levels (Fig. S1C and D), they failed to significantly alter tight junction protein expression or PLK2 levels (*P* > 0.05, Fig. S1E and F). These findings suggest that the heightened pathogenicity of HV *A. b* may be attributed, at least in part, to its capacity to specifically disrupt the PLK2-mediated protective axis, thereby compromising epithelial barrier integrity.

## DISCUSSION

Transcriptomics functions as a powerful tool for identifying crucial genetic responses to external perturbations via comparative analysis of phenotypic differences between experimental and control groups. It is essential for clarifying pathogenic mechanisms, guiding public health interventions, and formulating therapeutic strategies (19). However, the transcriptional profiles of cells infected with hypervirulent *A. baumannii* remain uncharacterized. To address this gap, we performed RNA sequencing on BEAS-2B cells infected with HV *A. b* to characterize the host transcriptional reprogramming. This study marks the initial endeavor to explore such transcriptional changes in the context of HV *A. b* infection. Integrated bioinformatics analysis revealed that PLK2 functions as a key regulator of *A. baumannii-*induced oxidative stress. Additionally, we identified the FoxO signaling pathway as a central node governing oxidative stress responses and epithelial barrier dysfunction, ultimately driving cellular demise upon HV *A. b* infection.

In *vitro*, hypervirulent *A. baumannii* concurrently suppresses PLK2 expression and impairs FoxO-mediated antioxidant defense via the downregulation of FoxO1 and FoxO3a, thereby driving epithelial barrier dysfunction in both BEAS-2B and HBE cells. This coordinated inhibition triggers excessive ROS accumulation and subsequent cellular damage. Notably, genetic or pharmacological inhibition of PLK2 exacerbated these effects, establishing PLK2 as a critical target for mitigating the pathogenicity of HV *A. b*. A notable advantage of this study is the novel transcriptomic analysis of infected cells. This analysis identified PLK2 as one of the most significant DEGs and confirmed the FoxO-mediated oxidative stress pathway as a key regulatory axis underlying the pathogenesis of hypervirulent strains. While PLK2 has been extensively studied in viral pathogenesis, its role in bacterial infections remains poorly defined. Proteomic analyses have shown that avian influenza virus exploits PLK2 to enhance its replication and induce apoptosis. Specifically, PLK2 silencing inhibits ROS production and P53-dependent apoptosis (12), a finding that contrasts with our observations. Conversely, Epstein-Barr virus employs the histone demethylase KDM5B to transcriptionally suppress PLK2, thereby facilitating viral persistence (20). These findings highlight a complex, context-dependent regulatory paradigm: viruses may promote PLK2 expression to evade host defenses and induce necrosis or inhibit it to drive pathogenesis. In the context of bacterial infections, miRNA-sequencing of largemouth bass infected with *Aeromonas hydrophila* identified an interaction between the FoxO signaling pathway and PLK2 within immune organs (21), suggesting an evolutionarily conserved axis. Extending these findings, our data demonstrate that *A. baumannii* orchestrates a coordinated suppression of this protective axis, simultaneously downregulating PLK2 expression, impairing FoxO-mediated antioxidant defenses (via FoxO1/FoxO3a degradation), and compromising epithelial barrier integrity. Notably, inhibition of PLK2 worsened all these outcomes, validating its role in the regulation of HV *A. b* pathogenicity.

Furthermore, the respiratory tract serves as a primary portal for pathogen entry. Airway epithelial cells, located at the environmental interface, rely on apical junctional complexes (AJCs)—comprising tight junctions (TJs) and adherent junctions (AJs)—to maintain epithelial integrity and preclude microbial invasion (22, 23). Disruption of these junctions, leading to elevated paracellular permeability, represents a critical early event in both bacterial and viral infections (24, 25). Consistent with this, our transcriptomic data revealed significant inhibition of genes related to the cytoskeleton and barrier function infected with hypervirulent *A. baumannii*. Mechanistically, we confirmed that PLK2 preserves epithelial integrity by restricting barrier hyperpermeability. This action restricts bacterial access to the underlying subepithelial tissues, thereby inhibiting deep-tissue invasion. Consequently, targeting PLK2 emerges as a promising strategy to mitigate barrier dysfunction and curb HV *A. b* pathogenicity.

Collectively, this study characterized transcriptional changes in HV *A. b*-infected BEAS-2B cells and demonstrated the role of PLK2 in modulating FoxO-mediated oxidative stress and cell barrier function. These findings provide a novel mechanistic framework for understanding pathogenesis and offer new strategies for adjuvant therapy aimed at attenuating the virulence of HV *A. b*. However, the current study primarily focused on the PLK2-FoxO1/FoxO3 axis during HV *A. b* infection, and the contributions of other stress-sensitive FoxO isoforms and canonical upstream pathways remain uncharacterized. Further investigation into these complementary pathways will refine our understanding of the molecular architecture governing host responses to hypervirulent pathogens.

## Data Availability

The raw RNA-seq data used in this study were obtained from our previous publication (18). The SRA project accession is PRJNA1510853. While the same datasetdata set was utilized, this study and the previous one addressed distinct biological question with independent analytical approaches, in compliance with standard research practices.

## SUPPLEMENTAL MATERIAL

Table S1 and Fig. S1 spectrum.00694-26-s0001.doc Cell viability under *A. baumannii* infection, and compares ROS levels and PLK2/barrier protein expression in BEAS-2B and HBE cells exposed to low-virulence strains.

ASM does not own the copyrights to Supplemental Material that may be linked to, or accessed through, an article. The authors have granted ASM a non-exclusive, world-wide license to publish the Supplemental Material files. Please contact the corresponding author directly for reuse.
