# Supplementary material for: Acinetobacter baumannii inhibits the PLK2/FoxO axis to induce oxidative stress and cell barrier dysfunction
Source: Microbiol Spectr. 2026 Aug 21;14(10):e00694-26. doi: 10.1128/spectrum.00694-26 (PMC13689406; doi:10.1128/spectrum.00694-26)
Supplement: Supplementary file 1 [file spectrum.00694-26-s0001.doc]

**Supplementary files**

**Table. S1**. Primer sequence of genes in cells

| Gene names | Sequence ( 5’-3’ ) |
| --- | --- |
| *PLK2* | F: TGCCCCCGGCGATCT  R: AATGGGGGCCTCCCTAGTAA |
| *FoxO1* | F:GCAGCCGCCACATTCAAC  R:CAGAACTTAACTTCGCGGGG |
| *FoxO3a* | F:CTCGAAGTGGAGCTGGACC  R:TCCTCGGGGATCATGGAGTC |
| *GAPDH* | F:GGTGAAGGTCGGAGTGAA  R:CAGAAGGGGCAGAGATGA |

**Figure S1.** (A) Alterations in the viability of BEAS - 2B cells subsequent to infection with bacterial suspensions at varying MOI. (B) Viability of BEAS - 2B cells infected by HV *A. b* at different time. (C) The effects of low virulent strains on ROS in BEAS-2B and HBE cells. (D) Quantitative results of ROS in HBE and BEAS-2B cells (n=3). (E) Effect of low virulent strains on the protein levels of PLK2, ZO-1, E-cadherin, and Occludin in BEAS-2B and HBE cells. (F) Quantitative results of the protein levels of PLK2, ZO-1, E-cadherin, and Occludin (n=3). ns: no significance. ** *P* < 0.01, *** *P* < 0.001, **** *P* < 0.0001.
